# Supplementary material for: Planktonic foraminifera organic carbon isotopes as archives of upper ocean carbon cycling
Source: Nat Commun. 2022 Aug 17;13:4841. doi: 10.1038/s41467-022-32480-0 (PMC9386020; doi:10.1038/s41467-022-32480-0)
Supplement: Supplementary file 1 — Supplementary Information [file 41467_2022_32480_MOESM1_ESM.pdf]

Supplementary information:

Age details of cores used in study

The first cm of each core was used for planktonic foraminifera test-bound $\delta^{13}C_{org}$ measurements. Two approaches were used to provide age estimates: AMS radiocarbon dates (RAPiD cores), and oxygen isotope stratigraphy, where benthic (bottom dwelling) or planktonic (surface water dwelling) foraminifera oxygen isotopes are compared with nearby dated and published records.

RAPiD core tops (0-1 and 0-0.5 cm)

The three RAPiD cores, RAPiD 06-3B (2228 m water depth, 0-1 cm), RAPiD 11-7B (2126 m water depth, 0-0.5 cm) and RAPiD 39-28B (2363 m water depth, 0.5-1.0 cm) were previously AMS C-14 dated using planktonic foraminifera carbonate (*Globigerina bulloides* for RAPiD 6-3B and RAPiD 11-7B, *Neogloboquadrina pachyderma* for RAPiD 39-28B) with ages of 707±35, 395±37 and 629±35 years respectively[1].

BOFS 14K (0-1 cm)

Ages from BOFS 14K come from benthic foraminifera (*Cibicidoides wuellerstorfi*) stable isotope stratigraphy and occurrence of the Vedde Ash (found at 26 cm dated around 12.152 ka[2,3]). Oxygen isotope values shown in the Figure below have likely been adjusted by adding 0.65‰ to resemble *Uvigerina* values. Lighter oxygen isotopes ($\delta^{18}O$) are representative of warmer, low ice volume climates. This suggests the interval 0 to 19 cm is Holocene (< 10 ka) in age.

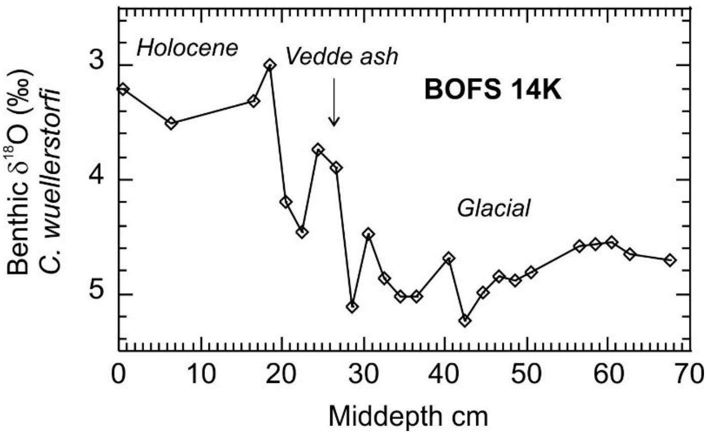

Supplementary Figure 1. Benthic foraminifera oxygen isotope record from Bertram et al.[2] for BOFS 14K plotted against depth in core. Approximate location of the Vedde Ash layer is indicated with the arrow.

IODP 303-306 U1308 (3871 meters water depth, 0-5 cm)

Epifaunal benthic foraminifera stable isotopes (monospecific *C. wuellerstorfi* or *C. mundulus*) were analysed for samples between 8 and 15 cm at this location, which consistently provided values between 2.48 and 2.87‰ indicative of Holocene values[4,5].

Depth in core (cm)	Benthic foraminifera species	$\delta^{18}O$ (‰)
8-9	*C. mundulus*	2.87
10-11	*C. wuellerstorfi*	2.48
12-13	*C. wuellerstorfi*	2.63
14-15	*C. wuellerstorfi*	2.68

Supplementary table 1. Stable oxygen isotopes of benthic foraminifera from IODP core U1308.

ODP 172 1057A (2584 meters water depth, 0-5 cm)

At ODP 1057A we measured oxygen isotopes of monospecific samples of the planktonic foraminifera *Globigerinoides ruber*, which with values between -1.14 and -1.51‰ indicate Holocene values[6,7].

Depth in core (cm)	Planktonic foraminifera species	$\delta^{18}O$ (‰)
6-7	*G. ruber*	-1.14
8-9	*G. ruber*	-1.3
10-11	*G. ruber*	-1.18
12-13	*G. ruber*	-1.16
14-15	*G. ruber*	-1.51

Supplementary table 2. Stable oxygen isotopes of planktonic foraminifera *G. ruber* at ODP Site 1057A..

ODP 177 1088C (2082 m water depth, 0-1 cm)

Ages at ODP 1088 come from planktonic foraminifera (*Globigerina bulloides*) stable isotope stratigraphy. Lighter oxygen isotope values, between 1.9 and 2.3‰ in the top 9 cm are indicative for Holocene values.

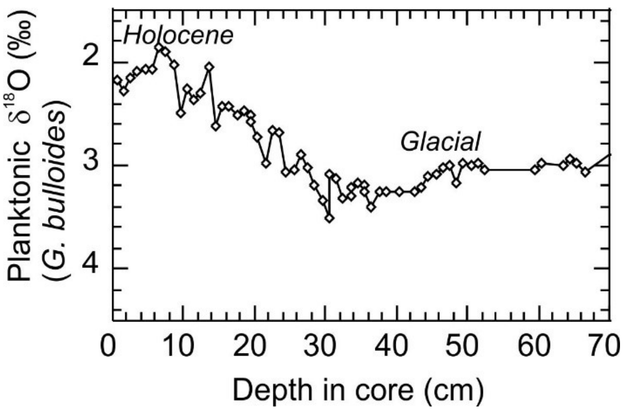

ODP 177 1090A (3702 m water depth, 0-2 cm)

Supplementary Figure 2. Planktic foraminifera (*G. bulloides*) oxygen isotope record for ODP 1088 plotted against depth in core.

At ODP 1090A, a *G. bulloides* $\delta^{18}O$ of 2.01‰ for the sample between 0-2 cm is indicative for the Holocene[8].

References

1. Kristjánsdóttir, Greta B; McCave, I Nick; Bryant, C (2011): AMS C-14 ages of coretops collected on RRS Charles Darwin cruise CD159, July 2004, N. Atlantic, for the NERC RAPID programme. Godwin Laboratory for Palaeoclimate Research, Department of Earth Sciences, University of Cambridge, Dataset #773254 (https://doi.org/10.1594/PANGAEA.773254).

2. Bertram, C.J., Elderfield, H., Shackleton, N.J., MacDonald, H.A., 1995. Cadmium/calcium and carbon isotope reconstructions of the glacial northeast Atlantic Ocean. *Paleoceanography* **10**, 563-578.

3. Manighetti, B., McCave, I.N., Maslin, M., Shackleton, N.J. 1995. Chronology for climate change: Developing age models for the Biogeochemical Ocean Flux Study cores. *Paleoceanography* **10**, 513-526, https://doi.org/10.1029/94PA03062.

4. Hoogakker, B.A.A., Downy, F., Andersson, M.A., Chapman, M.R., Elderfield, H., McCave, I.N., Lenton, T.M., Grützner, J., 2013. Gulf-Stream – substropical gyre properties across two Dansgaard-Oeschger cycles. *Quaternary Science Reviews* **81**, 105-113.

5. Thornally, D.J.R., Elderfield, H., McCave, I.N. 2010. Intermediate and deep water paleoceangraphy of the northern North Atlantic over the past 21,000 years. *Paleoceanography* **25**, PA 1211.

6. Keigwin, L.D., Schlegel, M.A., 2002. Ocean ventilation and sedimentation since the glacial maximum at 3 km in the western North Atlantic. *G-cubed* **3**, 10.1029/2001GC000283.

7. Hoogakker, B.A.A., Chapman, M.R., McCave, I.N., Hillaire-Marcel, C., Ellison, C.R.W., Hall, I.R., Telford, R.J., 2011. Dynamics of North Atlantic Deep Water masses during the Holocene. *Paleoceanography* **26**, PA4214.

8. Hodell, D.A., Charles, C.D., Curtis, J.H., Mortyn, P.G., Ninnemann, U.S., Venz, K.A., 2003. Data Report: Oxygen Isotope Stratigraphy of ODP Leg 177 Sites 1088, 1089, 1090, 1093 and 1094. In Gersonde, R., Hodell,D.A., and Blum, P. (Eds.), *Proc. ODP, Sci. Results*, **177**, 1–26.
